# High Prevalence of Tobacco Consumption among Pregnant Women in a Southern European City (Seville): A Challenge for the Health System

**DOI:** 10.3390/toxics12100728

**Published:** 2024-10-09

**Authors:** Ramón Mendoza-Berjano, Fatima Leon-Larios, Isabel Corrales-Gutierrez, Diego Gomez-Baya, Rocío Medero-Canela, Francisca Baena-Antequera

**Affiliations:** 1 Research Group on Health Promotion and Development of Lifestyle across the Life Span, University of Huelva, 21007 Huelva, Spain; ramon@dpsi.uhu.es (R.M.-B.); diego.gomez@dpee.uhu.es (D.G.-B.); 2 Department of Social, Developmental and Educational Psychology, University of Huelva, 21007 Huelva, Spain; 3 Nursing Department, Faculty of Nursing, Physiotherapy and Podiatry, University of Seville, 41001 Seville, Spain; fatimaleon@us.es; 4 Fetal Medicine Unit, University Hospital Virgen Macarena, 41009 Seville, Spain; icorrales@us.es; 5 Department of Surgery, Faculty of Medicine, University of Seville, 41001 Seville, Spain; 6 Huelva Costa Condado-Campiña Health District, Multidisciplinary Teaching Unit of Family and Community Care, 21005 Huelva, Spain; rocio.medero.sspa@juntadeandalucia.es; 7 Obstetric Unit, University Hospital Virgen de Valme, 41014 Seville, Spain; 8 Nursing Department, Osuna University School, 41640 Osuna, Spain

**Keywords:** prenatal tobacco exposure, pregnancy, smoking, sociodemographic correlates, obesity, prevention

## Abstract

The prevalence of prenatal tobacco exposure remains high in many countries, particularly in southern Europe. The aims of this study were to estimate the prevalence of smoking among pregnant women in a southern Spanish city (Seville) and to identify the associated sociodemographic and obstetric characteristics. In a descriptive, cross-sectional study, a random sample of pregnant women who were scheduled to undergo a morphology scan at their public referral hospital in their 20th week of gestation were interviewed in person. At the start of pregnancy, 38.2% of the pregnant women were smokers. In the twentieth week, 19.1% continued to smoke, and the same percentage had quit. The prevalence of smoking in pregnant women was higher among those with a low level of education (60% among pregnant women with no studies and 30.4% in those with primary education) and among those who had had abortions (38.5%). Pregnant smokers with obesity were the least likely to have given up smoking during pregnancy. Women with a lower educational level should be a prime target for cross-sectoral interventions aimed at preventing prenatal tobacco exposure. Implementation of support measures for providing effective clinical advice in preconception and prenatal care regarding healthy lifestyles is particularly needed.

## 1. Introduction

Since Simpson′s pioneering study on the impact of smoking during pregnancy on birth weight in 1957 [1], overwhelming evidence of the multiple risks of prenatal tobacco exposure (PTE) for both the child and the mother has been generated. Recent studies are contributing to a better understanding of the sequelae of tobacco use during pregnancy and are providing evidence on the mechanisms that explain the teratogenic action of PTE.

As far as the child is concerned, recent studies continue to corroborate that PTE is associated with fetal growth retardation, premature birth, and low birth weight, with a clear dose-effect relationship in terms of the number of cigarettes smoked [2,3,4,5,6,7,8]. In addition to the fetal hypoxia derived from the carbon monoxide inhaled by the mother when smoking and the neurotoxic and vasoconstrictive action of nicotine [9], PTE leads to various epigenetic changes. These changes have a dose-effect relationship, tend to be persistent, and are associated with delayed fetal development and low birth weight, among other conditions [3,10,11,12]. Similarly, PTE tends to cause reduced fetal heart rate and movements [13] and is also a significant risk factor for fetal mortality and miscarriage [7,14,15].

There is also evidence that PTE increases the risk of neonatal apnea, sudden infant death, congenital heart disease, neural tube defects, gastrointestinal or urogenital malformations, orofacial cleft, alterations in the immune system, infant asthma, allergic rhinitis, and visual problems in infancy and later childhood [7,16,17,18,19,20,21,22].

Moreover, neuroimaging studies in children and adolescents have shown that PTE alters brain development. It is associated with a significant reduction in the cortical gray matter, corpus callosum, hippocampus, and other limbic structures, as well as in the cerebellum [21,23]. In animal models, nicotine has been found to have teratogenic effects on the developing nervous system, causing neurotransmitter dysregulation and synaptic alterations, among other problems [24]. Recent research further demonstrates that in humans, prenatal nicotine exposure is associated with reduced head circumference and neurodevelopmental impairment [23]. In addition, maternal smoking during pregnancy is linked to difficulties in executive functions during childhood and adolescence [25]. PTE is also a risk factor for persistent disturbances in brain activity at rest, problems in speech development, reduced self-regulatory ability, attention and hyperactivity problems, delays in psychomotor development, and poorer academic development in childhood, as well as for persistent tic disorder [23,26,27,28,29,30,31,32,33].

Furthermore, PTE is associated with a predisposition to being overweight in childhood and adolescence [6,34,35]. It has also been found to be a predictor of health problems that may emerge in adulthood, such as hypertension or gallbladder disorders [36,37]. Another study suggests that it increases the perception of pain in neonates [38].

For pregnant women, smoking during pregnancy not only promotes the progressive onset of multiple diseases linked to tobacco use (e.g., breast cancer [39,40]). It is also a risk factor for health problems that can manifest during pregnancy, such as pelvic pain, placenta previa, ectopic pregnancy, sleep problems, preeclampsia, and stroke [41,42,43,44]. Additionally, it is a predictor of postpartum depression [45,46].

Given the mounting scientific evidence on the wide range of problems associated with tobacco use during pregnancy, a dramatic reduction in smoking prevalence in most countries would be expected. However, it is difficult to determine trends over time, as in many countries—including European ones [47]—such data are not regularly collected from representative samples of all pregnant women (not only from mothers who deliver healthy newborns or newborns without congenital defects) and with comparable methodology.

In the United States, between 2010 and 2017, a decrease in the prevalence of smoking during pregnancy was observed, but not among pregnant women aged 35–39 years; moreover, the trend showed an increase among less educated pregnant women [48]. The WHO European Region has the highest prevalence of tobacco use during pregnancy in the world, with an estimated rate of 8.1% for the region as a whole [49]. Within Europe, there are countries, such as Denmark, where there is a clear decrease in its prevalence, but the data show increasing social differences [50]. In Finland, smoking rates at the start of pregnancy remained fairly stable between 1991 and 2015 (around 15%) but increased in pregnant women under 25 years of age [51,52]. In Norway, Sweden, and Australia, the smallest decline in prevalence of smoking during pregnancy has been recorded in young female smokers [53]. In 2015, Norway, Sweden, and Lithuania reported rates of less than 5% prevalence among all pregnant women. By contrast, the Spanish region of Valencia, Wales, and France had rates above 15% [47]. Outside the European context, estimated rates of prevalence are generally lower than in Europe. For example, Japan reports a rate of 3.6% of women smoking in the second or third trimester of pregnancy [17].

In Spain, it has been estimated that smoking prevalence among pregnant women rapidly increased in the last two decades of the 20th century. It reached its highest level at the beginning of the 21st century and rapidly declined between 2002 and 2008. Since 2009, it has declined very slowly. It should be noted that in 2005 a law was enacted in Spain introducing several smoking prevention measures [54]. There also exists growing social inequality in this respect, with a sharp decline in smoking prevalence among university-educated pregnant women and an increase among low-educated pregnant women [55,56,57]. In 2016, the estimated prevalence for the country as a whole was 20.4%, a higher rate than that observed in 1980 (14.3%) [56]. Nevertheless, in the Spanish region of Catalonia there is a clear downward trend in prevalence rates, while in other regions prevalence is increasing or, more frequently, there are no data available to estimate the evolution of prevalence rates over time [48,58].

In short, tobacco use during pregnancy can be considered a significant public health problem with serious immediate and delayed consequences for the child, the mother, the family, and society as a whole. It is therefore necessary to intensify preventive efforts in this field, especially in countries with a high prevalence of this problem, such as Spain. Tobacco use during pregnancy is also a powerful factor in increasing or maintaining health and educational inequalities, as it mainly affects women from disadvantaged social sectors and their offspring [59]. A better understanding of the determinants and correlates of tobacco use during pregnancy would be useful to be able to adapt the interventions provided by healthcare professionals to the different groups of pregnant women according to their characteristics and to develop preventive actions aimed at society as a whole or specifically at the population groups most affected by this problem.

Considering the above, this study aimed to estimate the prevalence of pre-pregnancy smoking and the rate of current smoking in the 20th week of gestation. It also sought to identify the sociodemographic and obstetric characteristics associated with smoking before pregnancy, smoking cessation while pregnant, and continuation of smoking through the 20th week of gestation.

## 2. Materials and Methods

### 2.1. Study Design and Setting

In a descriptive, cross-sectional study, a representative sample of pregnant women scheduled to attend a morphology scan at their public referral hospital in a city in southern Spain (Seville) in their 20th week of gestation were interviewed in person.

### 2.2. Participants

The sample was randomly selected among pregnant women who attended the hospital′s outpatient clinics for the 20-week morphology scan. Every second pregnant woman who attended the hospital′s outpatient practices for this purpose during five consecutive months in 2016 (1664 in total) was asked to participate in this study. If one did not accept, the proposal was made to the next pregnant woman. The eligibility criteria for participation in this study were being approximately in the twentieth week of gestation, being able to speak and read Spanish fluently, and, once participation in this study had been accepted, signing the informed consent form. Eventually, 425 pregnant women agreed to participate, which represented a participation rate of 51.2%. A detailed description of the sample has been published elsewhere [60].

### 2.3. Data Collection Instrument and Variables

An ad hoc anonymous questionnaire designed by the research team was used, including mainly multiple-choice questions, together with some open-ended questions. The questionnaire was piloted prior to its use in this study to verify the ease of understanding of the questions and to optimize the response options. For the open-ended questions, the answer was written down verbatim by the interviewer and subsequently categorized by the research team.

### 2.4. Groups of Variables Included in the Questionnaire

Sociodemographic and somatometric variables included the following: age; educational level (categorized into four levels: no studies, primary education, secondary studies, and higher education); employment status (four values: full-time, part-time, unemployed, and other situations); relationship or marital status (received one of two values: (1) married or with a partner; or (2) separated, divorced, widowed, or without a partner); size of the population of residence; country of birth; height; and weight at week 20.

Obstetric variables included the following: age, age at first pregnancy; gravidity (i.e., number of pregnancies including the current one); vaginal deliveries; caesarean deliveries; miscarriages; abortions; problems with previous pregnancies or deliveries.

Variables related to care during pregnancy included the following: pregnancy planning; use of assisted reproductive techniques; trimester of awareness of pregnancy; pregnancy monitoring by health professionals; pregnancy follow-up at a high-risk clinic; and folic acid intake.

Variables related to tobacco use included the following: cigarette use before pregnancy; current smoking (including number of cigarettes, if smoked); quitting smoking in pregnancy; and comparison between pre-pregnancy and current smoking.

### 2.5. Field Work

The interviewer (a trained healthcare professional) contacted each randomly selected pregnant woman in the waiting room of the practice where the morphology scan was scheduled and asked her to participate in this study. If the woman accepted, the interview was conducted in an adjoining room. This fieldwork was carried out between March and July 2016.

### 2.6. Data Analysis

Following the quality control of the data recording, a univariate analysis of each variable was performed, and two new variables were generated: summary of tobacco use (with three values: (1) does not smoke since before pregnancy; (2) has stopped smoking during pregnancy; (3) continues smoking in the twentieth week) and body mass index (BMI), that was created using the women’s weight and height. Bivariate analyses were then performed to explore the possible association between smoking-related variables and each of the variables mentioned above (i.e., sociodemographic, somatometric, obstetric, care during pregnancy, and expectations of breastfeeding). For this purpose, Chi-squared tests and comparisons of means or analyses of variance were used as appropriate, and non-parametric tests (Mann–Whitney or Kruskal–Wallis tests) were used for variables without a normal distribution.

## 3. Results

### 3.1. Description of the Sample

With a mean age of 31.9 years, the age range of the 425 pregnant women in the sample was between 14 and 46 years. About 38% had primary studies, and a similar percentage had higher education. Approximately 39% worked full time, 28% were unemployed, and the rest were in other employment situations. In addition, 98% reported being married or having a partner. Moreover, 92% of the respondents were born in Spain. In terms of BMI, 52% had a normal weight, 29% had overweight, and 19% had obesity.

With regard to obstetric variables, 40.5% of the pregnant women were primigravidae; 35.5% had had vaginal deliveries, and 14.0% had had a caesarean section. Slightly more than one fifth (22.5%) had had a miscarriage, and 9.2% had had an abortion.

In about three out of four cases (74.5%), the current pregnancy had been planned. Pregnancies were diagnosed during the first trimester in almost the entire sample (96.7%). Regarding folic acid supplementation, almost all women reported to be taking folic acid (96.0%), mostly since the first trimester of pregnancy (60.5%).

### 3.2. Prevalence and Correlates of Smoking Prior to the Current Pregnancy

Of the pregnant women, 61.8% reported that they did not smoke before the start of their pregnancy, which means that 38.2% of them started their pregnancy as smokers (Table 1). The prevalence of pre-pregnancy smoking was particularly high among pregnant women aged 26–30 years (Table 2). A significant relationship was also observed in terms of educational level: the lower the educational level, the higher the rate of pre-pregnancy smoking (*p* < 0.0001; V = 0.21596). Specifically, while 72.5% of women with higher education reported being non-smokers before the onset of pregnancy, this percentage decreased to 30% among uneducated pregnant women (Table 3).

In terms of BMI, the group of women with overweight were most likely to report smoking before pregnancy (Table 4). Moreover, the prevalence of tobacco use prior to pregnancy was higher among those who reported having had an abortion. In addition, the prevalence of non-smoking before pregnancy was higher in the capital of the province (the city of Seville), but not much different between surrounding communities of different sizes (Table 4). No relevant differences were detected in smoking prior to pregnancy according to the other studied variables (i.e., sociodemographic, obstetric, or pregnancy care).

### 3.3. Tobacco Use Cessation during Pregnancy before Week 20

The rate of participants who reported having given up smoking during pregnancy (19.1%) was similar to that of those who reported smoking in the 20th week (19.1%). The 26–30 age group, which had the highest rate of pre-pregnancy tobacco use, was also the one with the highest rate of smoking cessation in the first 20 weeks of pregnancy (27.6%) (z = 2.4). Higher rates of smoking cessation during this period in small (rural) communities are shown, followed by the smoking cessation rates in Seville, and lower in pregnant women living outside of Seville in communities with >10,000 and >20,000 inhabitants (Table 4). The rate of smoking cessation among pregnant women did not significantly differ regarding other sociodemographic variables. As for BMI, pregnant smokers with obesity were the least likely to have given up smoking during pregnancy (Table 4). The variability in smoking cessation according to the obstetric or healthcare variables studied was irrelevant.

Most of the pregnant women who reported smoking at week 20 reported having reduced their tobacco use compared to before pregnancy (Table 1).

### 3.4. Prevalence and Correlates of Smoking in the Twentieth Week of Pregnancy

The prevalence of smoking at week 20 in the sample as a whole was 19.1%, as reported above. Relevant differences were detected in this regard according to age. Specifically, there was a relatively high rate in the younger age groups and a relatively low rate in the 31–35-year-old age group (Table 2). Moreover, the rate of pregnant smokers was inversely proportional to the level of education (Table 3). Regarding the size of the municipality, the prevalence of smoking at week 20 among pregnant women living outside the city of Seville in towns with more than 10,000 or 20,000 inhabitants approximately doubled the prevalence of those living in the city of Seville. Differences were also found according to BMI: smoking rates among pregnant women with obesity were higher than among women with normal weight (Table 4). Regarding obstetric variables, a higher prevalence of tobacco use was observed among women who reported having had an abortion (38.5%; *p* < 0.01). No relevant variability was observed according to the other obstetric variables explored or the type of health care received during pregnancy.

## 4. Discussion

The aim of this study was to estimate the prevalence and correlates of smoking before pregnancy and in the twentieth week of gestation, as well as smoking cessation during pregnancy, in a random sample of pregnant women living in a geographical area of southern Europe (Seville, Spain).

The standardized mean age of the sample was 31.9 years. This is very similar to the mean maternal age in Spain (32.0 years in 2016) [61], the country with the oldest maternal age in Europe [62].

The estimated prevalence of pre-pregnancy tobacco use was 38%, which would mean that, in this social context, prenatal tobacco exposure (PTE) occurs at the beginning of many pregnancies (approximately four out of ten). This is especially relevant, as the embryonic period is particularly vulnerable to teratogenic agents.

This prevalence of pre-pregnancy smoking was higher than that estimated in other neighboring European countries, such as France (29.8%) or Italy (20.5%), and considerably higher than that estimated in a Nordic country, such as Lithuania (8%) [47]. It was also higher than that estimated in northern Spanish regions such as Catalonia (22.8%) and Galicia (27.8%) [47,63]. In the Spanish region of Andalusia, which includes Seville, a study conducted with a sample of pregnant women in another province (Granada) showed a prevalence of 36%, close to that estimated in the present study in Seville [64]. This suggests that, in certain parts of Europe, there is still strong social pressure on women of childbearing age (and adolescent girls) to initiate and maintain tobacco use. It may also suggest that, in some countries, neither the healthcare system nor the education sector are using their full potential for professional action and social influence to discourage tobacco use, promote a nicotine-free lifestyle, and help young people who wish to give up smoking. The convergence of these factors contributes to PTE marking the start of many pregnancies.

The results of this study show a clear relationship between tobacco use prior to pregnancy and level of education, with a very high prevalence among women with no studies or with only primary studies. This is in line with the sociological evolution of the tobacco epidemic among the female population in Europe and in other industrialized high-income countries. In these countries, since the last decades of the 20th century, smoking has become a problem that mainly affects women from disadvantaged social sectors [16,65,66].

The results also show that pre-pregnancy tobacco use is particularly prevalent among pregnant women aged 26–30 years, who were in the adolescent cohort at the beginning of the 21st century. A nationwide study conducted in Spain in 2002 estimated a 46.2% prevalence of tobacco use among 16-year-old adolescent female students [67]. A relationship can be inferred between the high prevalence of smoking among Spanish adolescent girls at the beginning of the 21st century and the high rate of pre-pregnancy smoking among the pregnant women of the study sample 14 years later.

Approximately one fifth of pregnant women (19.1%) in the present study stopped smoking when they were already pregnant. This rate is similar to that found in a Portuguese study of pregnant women in the third trimester (19%) [68] and slightly higher than that of pregnant women in the Spanish region of Galicia who stopped smoking on their own in the first trimester (12.3%) [69].

In this study, the 26–30 age group was the one with the highest rates of both pre-pregnancy smoking and smoking cessation while pregnant. It cannot be stated, therefore, that pregnant women aged 26–30 years have greater difficulty giving up smoking than those in the other age groups.

In this sample of pregnant women in Seville, no relevant differences were observed during pregnancy in smoking cessation depending on the level of education. This differs from the findings of research carried out in other contexts, which showed a higher rate of smoking cessation among women with a higher level of education [69,70]. Similarly, other studies have found that primigravidae are more likely to give up smoking [69,71], while the present study found no significant variability according to gravidity. Interestingly, the smoking cessation rate was found to be very low among pregnant women living with obesity (9.1%), which could indicate that this sector of pregnant women finds little support to give up smoking. Further studies should explore to what extent this is related to the social stigmatization of obesity, which may also affect healthcare itself [72]. Results suggest that it would be advisable for the healthcare system to adopt measures that make it easier for healthcare professionals to provide respectful, warm, and effective help to pregnant smokers and, in particular, to pregnant women with obesity, so that they can overcome their dependence on nicotine.

The prevalence of smoking among the pregnant women in the sample (all in approximately the 20th week of gestation) was 19.1%. This rate was significantly higher than that estimated in studies conducted in other areas with women in the second trimester of pregnancy, such as Denmark (7.5%) [47] or Shanghai, China (0.9%) [8].

Although Europe stands out globally for its high rates of tobacco use during pregnancy [49], these vary considerably within the region. As noted above, countries such as Norway, Sweden, and Lithuania have achieved prevalence rates below 5% [47]. In general, these are the same countries that have also achieved a drastic reduction in smoking rates in the general population, among women of childbearing age, and in the adolescent population. In fact, between 2000 and 2010, northern European countries were found to be the most successful in promoting smoking cessation among both men and women, which calls for intensified preventive efforts in the other geographical areas of the European region [73].

Some studies show an interrelation between smoking prevention in the general population and reduced smoking prevalence among pregnant women [74,75]. This may indicate that smoking prevention measures aimed at the general population also seem to benefit women of childbearing age, in particular pregnant women. They have also been found to have beneficial effects on infant health and reduce neonatal mortality [76,77,78].

Unfortunately, in all European regions, there was an increase in smoking initiation in early adolescence (11–15 years) among adolescent girls between 1990 and 2009 [79]. However, some countries, such as Iceland, are being particularly exemplary in their achievements in reducing smoking rates among adolescent girls and boys [80,81].

In the present study, significant differences were found in the prevalence of pregnant smokers according to educational level; it was particularly high among women with no studies or with only primary studies. This is consistent with the findings of research conducted in other countries, such as the United States, Denmark, and Norway [50,82,83], and in Spain [56,63,84,85]. This higher prevalence of smoking among pregnant women from disadvantaged social groups may constitute a risk factor that acts in synergy with other factors that are also more frequent among this population group, thus reinforcing health inequalities among pregnant women [50]. Through its immediate and delayed impact on child development, PTE is, in turn, a factor that increases social inequality in the health and cognitive development of new generations. Consequently, women from disadvantaged social sectors should be a priority target for interventions carried out in pre-pregnancy and prenatal care by healthcare systems. In addition, intersectoral public policies are needed to provide all pregnant and postpartum women with access to the material and social resources they may need so that at least the first 1000 days of life (from the start of pregnancy) can be spent in adequate conditions [86,87]. At the same time, evidence shows that certain tobacco control measures, such as increasing the price of tobacco products through higher taxation and providing cessation programs particularly targeted at smokers from disadvantaged social groups, contribute to reducing social differences in tobacco consumption [88,89].

Outstanding differences in the prevalence of smoking at week 20 among pregnant women according to size of municipality have been found in this study. It was particularly high among those women living outside the city of Seville in towns with more than 10,000 or 20,000 inhabitants. Agriculture and industry are the main economic activities in these towns, where in addition the percentage of women with higher degrees is lower than in the city of Seville.

If the pregnant woman’s partner smokes, it is advisable to discuss tobacco use with that person as well. Having a smoking partner increases the likelihood that the pregnant woman will continue smoking [64,90]. It may also be a source of environmental tobacco smoke in the home or be a risk factor for heritable problems in their future offspring [91].

It is of particular importance to raise social awareness of the need to avoid smoking in the presence of pregnant women to prevent exposure of pregnant women to second-hand smoke in the home, work, or leisure environments [92,93]. Exposure to environmental tobacco smoke can also be teratogenic. It increases the risk of miscarriage [94], preterm birth [31], low birth weight [8], neural tube defects, and other birth defects [95,96]. Moreover, it affects neurodevelopment in early childhood, potentially leading to impaired psychomotor, language, and cognitive development [97,98]. It also favors the occurrence of rhinitis [21] and increases the risk of childhood obesity [6].

Non-combustible nicotine products may be perceived as a safe alternative to cigarette smoking, but they are also a risk [99,100]. Prenatal nicotine exposure impairs cardiorespiratory function, learning and memory, executive functions, and brain reward circuits and causes permanent changes in the genome that can be inherited [23]. Gestational nicotine exposure causes a wide range of alterations to brain development and can contribute to the development of ADHD, schizophrenia, anxiety, obesity, and future adolescent substance abuse. This shows the importance of stopping smoking and any form of nicotine exposure during pregnancy to mitigate the risk of long-lasting complications in offspring [101].

Several qualitative studies conducted in high-income countries have shown that a relevant sector of pregnant women finds barriers to giving up smoking or alcohol consumption; some of them are unresponsive health professionals, lack of information and dialogue about the risks of these behaviors, and lack of social support. By contrast, some of them report awareness of the risks of substance use, having intrinsic incentives, and finding support from family, friends, and professionals as supportive elements [102,103]. Professionals may face organizational barriers or training deficits that hinder them from effectively assisting pregnant women in adopting or maintaining a healthy lifestyle during pregnancy [104,105]. Therefore, healthcare systems should prioritize research on the barriers encountered by professionals in the prevention of prenatal exposure to tobacco or alcohol, with a view to articulating institutional programs that enhance and facilitate appropriate professional practice in this field. Moreover, a Swedish study identified that women are less frequently asked and counseled about tobacco consumption by primary healthcare professionals than men [106]. This suggests the advisability of exploring whether such biases in healthcare professional practice also exist in other countries. The effectiveness of midwifery group practice when working with particularly vulnerable women should also be assessed [107]. In addition, synergies between healthcare systems and social services should be facilitated in the care of pregnant and postpartum women from disadvantaged social groups. The quality of the healthcare provided to adolescent pregnant women may also need to be assessed [108].

In Spain, preconception care is not systematically promoted, and this may also play a role in the high prevalence of tobacco use among pregnant women in Spain. Access to this preconception healthcare assessment is low. This is because it is not available in all reference health centers and because a large sector of the female population does not find it necessary or believes that the information obtained by other means is sufficient [109]. This is the case despite the proven effectiveness of interventions aimed at improving women’s pre-pregnancy habits [110,111]. Just as in the preconception medical visit, there is evidence of the effectiveness of interventions to improve lifestyles during adulthood in the workplace [112] that could be useful for promoting healthy lifestyles among women of childbearing age.

This study has a number of strengths but also certain limitations. One of the strengths is the random selection of the sample from among the pregnant women receiving care at the reference hospital of their health area. The interviews were conducted in person at the same health center, using an anonymous questionnaire and in a context that facilitated truthful answers. Moreover, all the pregnant women were in approximately the twentieth week of gestation, so the sample was homogeneous in this respect. The mean age of the sample coincided with the mean age of motherhood in Spain.

One of the limitations of this study is its descriptive cross-sectional nature, which does not allow establishing causal relationships between the studied variables. Pregnant women who did not understand Spanish were excluded from the sample, as interpreters were not available. In addition, the variables describing tobacco use were measured with self-reported consumption habits, that is, excluding the use of biomarkers such as the determination of cotinine in saliva and urine or the measurement of exhaled carbon monoxide. Thus, the validity of the data may be questioned. However, evidence from other studies shows a good correlation between self-reported consumption and cotinine levels [113,114]. Furthermore, it was assumed that no participant became a smoker after the time of conception, which could be another limitation.

## 5. Conclusions

Tobacco consumption during pregnancy, in addition to compromising maternal health, entails serious and potentially long-lasting risks to child development. As this issue is particularly prevalent among pregnant women with obesity or a lower educational level, it contributes to reinforcing health inequalities. Moreover, as prenatal exposure to tobacco can also affect the cognitive development of children, the fact that offspring of less-educated women are more likely to be affected by this exposure contributes to persisting inequalities as regards educational opportunities. Tobacco consumption during pregnancy can be prevented, as evidenced by the fact that some countries in Europe and other geographical areas have managed to reduce it to minimum levels. Therefore, the high prevalence identified in this random sample of pregnant women in a southern European city (Seville) illustrates the need to adopt effective preventive measures both by the healthcare system and other sectors (in particular, education and social services). Similarly, research into the barriers encountered by healthcare professionals in promoting healthy lifestyles in preconception and prenatal care could serve as a basis for establishing measures across the healthcare system that stimulate and enable the adoption of this approach in healthcare.

## Figures and Tables

**Table 1:** Tobacco consumption of pregnant women who participated in this study.

Variables	Categories	No.	%
Tobacco consumption (overview)	No smoking since before pregnancy	262	61.8
	Smoking cessation being pregnant	81	19.1
	Does smoke (in the 20th week of gestation)	81	19.1
Tobacco consumption compared to before pregnancy	Did not smoke before pregnancy	262	61.8
	Did smoke, as now	12	2.8
	Did smoke, but has given up	81	19.1
	Did smoke, more than now	67	15.8
	Did smoke, less than now	2	0.5
Cigarettes smoked	1–3 cigarettes a day	11	2.6
	4–6 cigarettes a day	14	3.3
	7–10 cigarettes a day	16	3.8
	11–14 cigarettes a day	3	0.7
	15–20 cigarettes a day	3	0.7
	Sporadically throughout the week	2	0.5
	Sporadically throughout the month	1	0.2
	Does not smoke	375	88.2

**Table 2:** Tobacco consumption of pregnant women who participated in this study according to age.

Variables	Categories	Younger Than 25		From 26 to 30 Years		From 31 to 35 Years		Older Than 35		Sign.
		*N*	%	*N*	%	*N*	%	*N*	%	
Tobacco consumption (overview)	No smoking since before pregnancy	31	58.5	45	45.9	111	68.9	75	67.0	<0.01
	Smoking cessation being pregnant	8	15.1	27	27.6	28	17.4	18	16.1	V = 0.14518
	Does smoke (in the 20th week of gestation)	14	26.4	26	26.5	22	13.7	19	17.0	
Tobacco consumption compared to before pregnancy	Did not smoke before pregnancy	31	58.5	45	45.9	111	68.9	75	67.0	<0.05
	Did smoke, as now	0	0.0	4	4.1	4	2.5	4	3.6	V = 0.137
	Did smoke, but has given up	8	15.1	27	27.6	28	17.4	18	16.1	
	Did smoke, more than now	14	26.4	21	21.4	18	11.2	14	12.5	
	Did smoke, less than now	0	0.0	1	1.0	0	0.0	1	0.9	
Cigarettes smoked	1–3 cigarettes a day	4	7.5	4	4.0	2	1.2	1	0.9	<0.05
	4–6 cigarettes a day	2	3.8	5	5.1	3	1.9	4	3.6	V = 0.16632
	7–10 cigarettes a day	5	9.4	6	6.1	2	1.2	3	2.7	
	11–14 cigarettes a day	0	0.0	2	2.0	1	0.6	0	0.0	
	15–20 cigarettes a day	0	0.0	1	1.0	2	1.2	0	0.0	
	Sporadically throughout the week	0	0.0	1	1.0	1	0.6	0	0.0	
	Sporadically throughout the month	1	1.9	0	0.0	0	0.0	0	0.0	
	Does not smoke	41	77.4	80	80.8	150	93.2	104	92.9	
Tried to give up smoking being pregnant	No	1	5.0	6	16.7	5	17.2	2	7.7	
	Yes	19	95.0	30	83.3	24	82.8	24	92.3	

**Table 3:** Tobacco consumption of pregnant women who participated in this study according to educational level.

Variables	Categories	No Studies		Primary Education		Secondary Studies		Higher Education		Sign.
		*N*	%	*N*	%	*N*	%	*N*	%	
Tobacco consumption (overview)	No smoking since before pregnancy	6	30.0	162	50.3	124	66.7	232	72.5	<0.0001
	Smoking cessation being pregnant	2	10.0	62	19.3	42	22.6	56	17.5	V = 0.21596
	Does smoke (in the 20th week of gestation)	12	60.0	98	30.4	20	10.8	32	10.0	
Tobacco consumption compared to before pregnancy	Did not smoke before pregnancy	3	30.0	81	50.3	62	66.7	116	72.5	<0.0001
	Did smoke, as now	0	0.0	9	5.6	0	0.0	3	1.9	V = 0.19039
	Did smoke, but has given up	1	10.0	31	19.3	21	22.6	28	17.5	
	Did smoke, more than now	6	60.0	39	24.2	10	10.8	12	7.5	
	Did smoke, less than now	0	0.0	1	0.6	0	0.0	1	0.6	
Cigarettes smoked	1–3 cigarettes a day	2	20.0	7	4.3	1	1.1	1	0.6	<0.0001
	4–6 cigarettes a day	2	20.0	10	6.2	1	1.1	1	0.6	V = 0.22735
	7–10 cigarettes a day	2	20.0	10	6.2	3	3.2	1	0.6	
	11–14 cigarettes a day	0	0.0	3	1.9	0	0.0	0	0.0	
	15–20 cigarettes a day	0	0.0	2	1.2	0	0.0	1	0.6	
	Sporadically throughout the week	0	0.0	2	1.2	0	0.0	0	0.0	
	Sporadically throughout the month	0	0.0	1	0.6	0	0.0	0	0.0	
	Does not smoke	4	40.0	126	78.3	89	94.7	156	97.5	
Tried to give up smoking being pregnant	No	0	0.0	8	12.7	2	12.5	4	15.4	
	Yes	6	100.0	55	87.3	14	87.5	22	84.6	

**Table 4:** Association between smoking during pregnancy and sociodemographic and somatometric variables.

Variables/Categories	Did Not Smoke Since before Pregnancy	Smoking Cessation Being Pregnant	Does Smoke (in the 20th Week of Gestation)	Statistical Parameters
Employment status	*N* = 261 %	*N* = 81 %	*N* = 81 %	Chi^2^ (6) = 6.40; *p* = 0.38; V-Cramer = 0.087
Full-time	66.5	17.4	16.2	
Part-time	64.8	18.5	16.7	
Unemployed	60.7	20.5	18.8	
Other situations	51.8	21.2	27.1	
Size of population of residence	*N* = 262 %	*N* = 81 %	*N* = 81 %	Chi^2^ (6) = 13.04; *p* = < 0.05; V-Cramer = 0.124
Up to 10,000 inhabitants	54.1	24.3	21.6	
From 10,001 to 20,000 inhabitants	55.3	13.2	31.6	
More than 20,000 hab. except for the capital	53.8	12.8	33.3	
Capital (Seville)	64.5	20.0	15.5	
Marital status	*N* = 262 %	*N* = 81 %	*N* = 81 %	Chi^2^ (2) = 2.37; *p* = 0.31; V-Cramer = 0.075
With a partner	62.3	19.0	18.8	
Without a partner	37.5	25.0	37.5	
Country of origin	*N* = 260 %	*N* = 81 %	*N* = 81 %	Chi^2^ (2) = 3.37; *p* = 0.19; V-Cramer = 0.089
Spain	60.4	19.5	20.1	
Other	75.8	15.2	9.1	
Body Mass Index	*N* = 258 %	*N* = 80 %	*N* = 78 %	Chi^2^ (6) = 16.11; *p* = < 0.05; V-Cramer = 0.139
Underweight	0.0	0.0	100.0	
Normal	67.0	18.8	14.2	
Overweight	53.3	25.0	21.7	
Obesity	62.3	11.7	26.0	

## Data Availability

The datasets presented in this article are not readily available due to privacy restrictions. Requests to access the datasets should be directed to the corresponding author.
